# Synergistic Effect of Fullerenes on the Laser-Induced Periodic Surface Structuring of Poly(3-Hexyl Thiophene)

**DOI:** 10.3390/polym11020190

**Published:** 2019-01-22

**Authors:** Álvaro Rodríguez-Rodríguez, Edgar Gutiérrez-Fernández, Mari-Cruz García-Gutiérrez, Aurora Nogales, Tiberio A. Ezquerra, Esther Rebollar

**Affiliations:** 1 Instituto de Estructura de la Materia (IEM-CSIC), Serrano 121, 28006 Madrid, Spain; alvaro.rodriguez@iem.cfmac.csic.es (Á.R.-R.); edgar.gutierrez@iem.cfmac.csic.es (E.G.-F.); maricruz@iem.cfmac.csic.es (M.-C.G.-G.); aurora.nogales@csic.es (A.N.); t.ezquerra@csic.es (T.A.E.); 2 J. Heyrovsky Institute of Physical Chemistry, ASCR, Dolejskova 3, Prague, Czech Republic; 3 Instituto de Química Física Rocasolano (IQFR-CSIC), Serrano 119, 28006 Madrid, Spain

**Keywords:** laser nanostructuring, poly(3-hexyl thiophene), atomic force microscopy, conducting-atomic force microscopy

## Abstract

Ordered and homogeneous laser-induced periodic surface structures (LIPSS) could be fabricated in poly(3-hexyl thiophene):[6,6]-phenyl C71-butyric acid methyl ester (P3HT:PC_71_BM) blends by using wavelengths in the ultraviolet (UV) range (266 nm). The absorption coefficient of PC_71_BM, which is maximum in its UV–Visible absorption spectrum around 266 nm, enhanced the overall absorption of the blend. In addition, PC_71_BM itself was capable of developing homogeneous LIPSS by laser irradiation at λ_laser_ = 266 nm. Therefore, we proposed that the synergistic effect of PC_71_BM on the LIPSS formation in P3HT:PC_71_BM (1:1) was due to a templating effect for the LIPSS formation of the PC_71_BM itself, which added to the overall increment of the absorption of the blend. LIPSS formation at ambient conditions in this wavelength range led to chemical modification of both P3HT and PC_71_BM, which rendered to non-conducting samples. Irradiation in vacuum significantly reduced radiation damage, rendering to the characteristic electrical conductivity pattern observed in P3HT LIPSS samples irradiated in the visible range. This effect could be of potential interest in order to obtain LIPSS in low absorbing polymers.

## 1. Introduction

**Figure 1:**
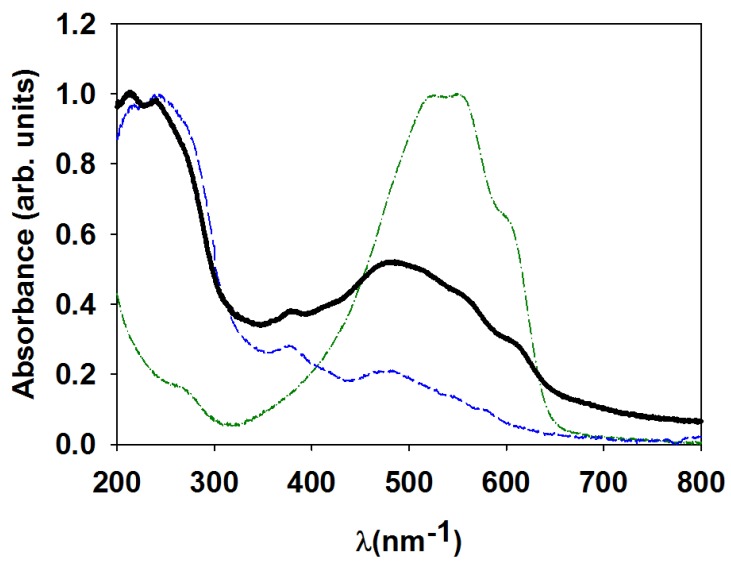
UV–Visible spectra of poly(3-hexyl thiophene) (P3HT, green dot-dashed line), [6,6]-phenyl C71-butyric acid methyl ester (PC_71_BM, blue dashed line) and P3HT:PC_71_BM (black line) thin films, deposited on quartz substrates.

Polymer surface nanostructures are gaining interest as functional elements for a broad range of applications, ranging from organic electronics [1] to self-cleaning [2] and energy harvesting [3], among others. The fabrication of nanostructured polymer surfaces remains in general, a challenge depending on the functionality it is aimed for. In addition to conventional lithographic methods, alternative procedures that simplify polymer nanostructuring are highly welcome, since the need for clean rooms, mask fabrication and complex equipment can be a burden, which is difficult to overcome in some cases. Use of laser-induced periodic surface structures (LIPSS) is a versatile method to create patterns in a great variety of materials, including polymers [4]. LIPSS in polymers appear by illumination with a pulsed laser of a solid smooth surface. Laser pulses in the range from nanoseconds to femtoseconds, with wavelengths from visible to infrared (IR), have been reported to produce LIPSS in polymers [4,5]. Typical LIPSS are grooves consisting of periodic hills and valleys, with pitches close to the wavelength of the laser and depths in the tens to hundred nanometer range [6,7,8]. By controlling laser polarization, more complex structures have also been described [9]. The mechanism of LIPSS formation is proposed to be due to the interference of the incoming laser with the wave scattered by the surface accompanied by a feedback effect [10]. Among semiconducting polymers, poly(3-hexyl thiophene) (P3HT) is still attracting significant attention due to its potential applications in optics, electronics and photovoltaics [11]. P3HT is a semi-crystalline polymer belonging to the poly(alkyl thiophene) family, which due to the presence of the alkyl side chains, can be easily processed either by melting or by solution [12,13]. Nanostructuring by laser in P3HT and in the all-polymer heterojunction P3HT/PCDTBT has been recently accomplished, obtaining functional electrically-conducting nanostructured surfaces [14,15,16]. By using nanosecond laser pulses with a wavelength in the visible (532 nm) and in the ultraviolet (266 nm) range, periodic ripples in P3HT have been prepared by LIPSS, with periods of about 475 and 280 nm, respectively [15]. However, while irradiation with visible pulses renders homogeneous and regular grating-like morphology, irradiation with UV pulses produces rather defective LIPSS with a lower level of ordering [15]. This effect was attributed in a first approach to the lower absorption coefficient of P3HT at 266 nm, than at 532 nm (Figure 1). It is worth mentioning that LIPSS P3HT films irradiated at 532 nm exhibit a characteristic heterogeneous electrical conductivity profile, consisting of conducting valleys and non-conducting hills. On the basis of Raman spectroscopy and grazing-incidence wide-angle X-ray scattering experiments, it was proposed that lower electrical conductivity of the hills can be attributed to a decrease of the crystallinity, induced by quick quenching after laser-induced melting of the polymer [15]. On the contrary, LIPSS P3HT samples irradiated at 266 nm are comparatively non-conducting, suggesting chemical damage [15]. Total electron yield (TEY) from near-edge X-ray absorption fine structure (NEXAFS) spectroscopy experiments showed that the spectrum of the LIPSS P3HT sample irradiated at 266 nm, prepared at ambient conditions, exhibited significant variations in comparison with that of the non-irradiated one, which indicated significant chemical bond modifications [17].

Considering that miniaturization is one of the driving forces pulling science and technology toward the limits of both materials and devices performance [18], it seems appropriate to optimize LIPSS at shorter wavelengths. One way to deal with this issue is to add more efficient absorbing additives to the polymer in order to increase the overall absorption coefficient of the material. As far as P3HT is concerned [6,6]-phenyl C71-butyric acid methyl ester (PC_71_BM) is an appropriate candidate for this purpose, since it has a maximum in its UV–Visible (UV–Vis) absorption spectrum in the 266 nm range [17]. Blends of P3HT and fullerenes have received significant attention due to their photovoltaics applications [19]. LIPSS in P3HT:PC_71_BM, irradiated at visible wavelengths, have been previously reported to exhibit photovoltaic properties [20]. 

In this paper we report on the synergistic effect of PC_71_BM in the LIPSS formation on P3HT: PC_71_BM at UV wavelengths. By using a combination of techniques based on NEXAFS and atomic force microscopy (AFM), we probe that regular and homogeneous LIPSS can be formed in P3HT:PC_71_BM by irradiation in the UV range (λ = 266 nm), overcoming the low absorption of P3HT in this wavelength range. Moreover, we demonstrate that in addition to the improvement of the overall absorption obtained in P3HT:PC_71_BM by incorporating the additive, the fullerene itself experiences an efficient nanostructuring upon irradiation at this wavelength.

## 2. Materials and Methods

P3HT (Ossila, *M*_w_ = 34,100 g/mol, PDI = 1.7, regioregularity = 94.7%) and phenyl C_71_-butyric acid methyl ester (PC_71_BM, Ossila (Sheffield, UK)) were mixed in chlorobenzene solutions (24 g/L) and stirred at 30 °C for 1 hour. Either P3HT or P3HT:PC_71_BM blends (weight ratio of 1:0 and 1:1, respectively) were deposited on n-silicon (100, Arsenic dopant, ACM (Lannion, France)) and then spin-coated at 2400 rpm for 120 s. The wafers were previously cleaned with acetone and isopropanol. Thin films of about 140 ± 20 (P3HT) and 120 ± 10 nm (P3HT:PC_71_BM) in thickness, with a roughness of a few nanometers, can be obtained by this method.

PC_71_BM was dissolved (40 mg/mL) in chlorobenzene and stirred at room temperature for 60 min. Precise amounts of fullerene solution (0.2 mL) were dropped by a syringe on a square n-silicon (100, Arsenic dopant, ACM (Lannion, France)) substrate, placed in the center of a metallic horizontal plate. A rotation rate of 2400 rpm was applied for 120 s. Thin films of ≈100 nm thickness can be obtained in a reproducible manner by this procedure. 

Near-edge X-ray absorption fine structure (NEXAFS) and resonant soft X-ray scattering (RSoXS) spectroscopy experiments were performed in the soft X-ray scattering beamline (11.0.1.2) at the Advanced Light Source of Lawrence Berkeley National Laboratory (Berkeley, CA, USA) as previously described [17,21]. For NEXAFS measurements, films were deposited by spin coating on silicon substrates with a previous spin-coated layer of poly(3,4-ethylenedioxythiophene):polystyrene sulfonate (PEDOT:PSS). Since PEDOT:PSS is water soluble, the polymer/fullerene blend films can be separated from the substrate by their immersion in water. Floating films were picked up with silicon nitride membranes. 

A linearly polarized laser beam delivered by a Q-switched Nd:YAG laser (Lotis TII LS-2131M, Minsk, Belarus), with a pulse duration of 8 ns was used. Laser irradiation was carried out in ambient conditions at normal incidence. The fourth laser harmonic (λ_laser_ = 266 nm) at a repetition rate of 10 Hz was used. The irradiation fluences were estimated by measuring the laser energy in front of the sample, considering 5 mm as the diameter of the laser spot. For the experiments performed in the PC_71_BM films, a 1.2 mm diameter iris was employed. 

Atomic force microscopy (AFM) measurements were accomplished by using a scanning probe microscope (MultiMode 8 equipped with a C-AFM module and with the Nanoscope V controller, Bruker, Karlsruhe, Germany). The topography AFM images were collected in tapping mode using silicon probes (NSG30 probes by NT-MDT, Moscow, Russia). For the electrical measurements by C-AFM conductive tips (Pt-Ir covered Si probes with a low spring constant, k = 0.2 Nm^−1^, SCM-PIC by Bruker) were used in contact mode. The sample was partially glued with a conductive silver epoxy (CW2400, Chemtronics, Hoofddorp, The Netherlands) on an iron support. In C-AFM measurements the conducting tip acts as a nanoelectrode contacting the sample surface. A bias voltage was applied between the cantilever and the conducting substrate. 

UV–Vis absorption spectra were acquired using a UV-3600 Shimadzu Spectrophotometer (Duisburg, Germany) on thin films deposited on quartz substrates. 

## 3. Results

### 3.1. Laser-Induced Periodic Surface Structuring on P3HT at 266 nm

**Figure 2:**
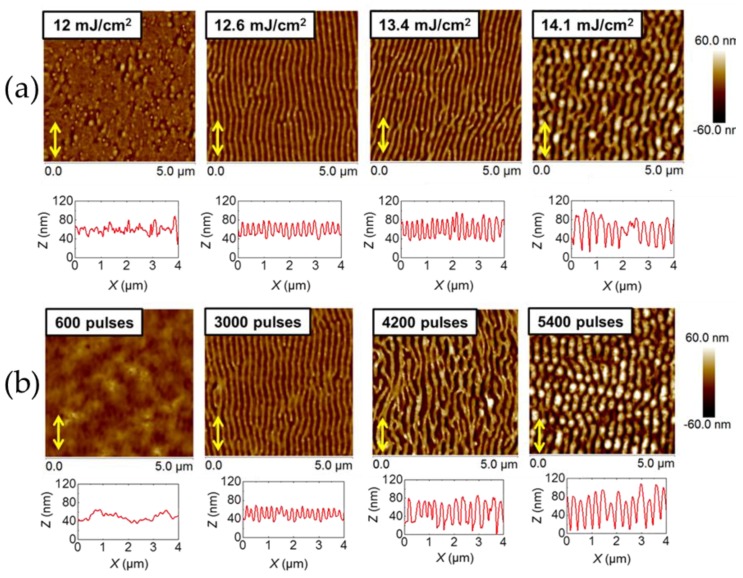
Atomic force microscopy (AFM) topography images of laser-induced periodic surface structures (LIPSS) on P3HT thin films, at an irradiation wavelength of 266 nm at: (**a**) 3600 pulses varying the fluence and at (**b**) 13.4 mJ/cm^2^ varying the number of pulses. Horizontal height profiles over lengths of 4 µm are shown at the bottom of every image. The double arrow (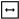) indicates the polarization vector of the laser beam.

**Figure A1:**
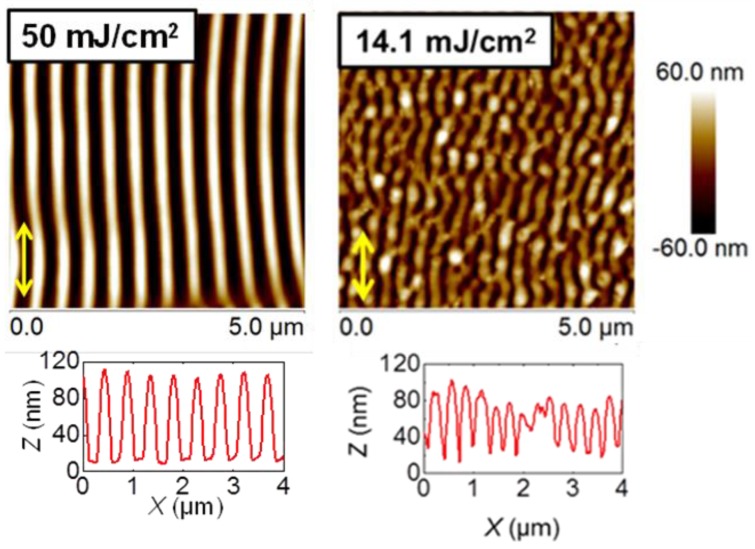
Comparative AFM height images of LIPSS obtained on P3HT thin films with 3600 pulses at 532 nm (fluence of 50 mJ/cm^2^) and at 266 nm, fluence of 14.1 mJ/cm^2^. Horizontal height profiles over lengths of 4 µm are shown at the bottom of each image. The polarization vector of the laser beam is indicated by double arrows (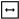).

Figure 2 shows topography images of LIPSS fabricated at ambient conditions, exhibiting different periods and heights. They were obtained at 266 nm, at a fixed number of pulses of 3600 and different fluences (Figure 2a), and at a fixed fluence of 13.4 mJ/cm^2^ and different number of pulses (Figure 2b). A systematic description of periods and heights dependence with both fluence and number of pulses has been published elsewhere [15]. In brief, the period increased up to a maximum for a value of fluence of about 14 mJ/cm^2^ and then remained practically constant at a value close to the irradiation wavelength, while the depth of ripples increased up to 85 nm for 14 mJ/cm^2^. Above 14.7 mJ/cm^2^ the ripples started to deteriorate, due to ablation (Figure 2a). Similar behavior was observed (Figure 2b) when fabricating LIPSS at a fixed fluence of 13.4 mJ/cm^2^ and a different number of pulses. Here, the period remained constant for about 2500 pulses and subsequently increased, reaching a plateau with a value close to the laser wavelength upon irradiation with 5500 pulses. The variation of LIPSS depth as a function of the number of pulses followed a similar behavior, increasing up to 5000 pulses and reaching a plateau of about 70 nm. The results shown in Figure 2 reveal that in general, LIPSS fabricated at 266 nm presented structures with a lower degree of order than those created with the irradiation wavelength of 532 nm [15]. For the sake of comparison, Figure A1 in the Appendix A compares typical LIPSS obtained in P3HT by laser irradiation at 532 and 266 nm for conditions of fluence and pulses in which the optimal structures appear for each wavelength [15]. Differences in the quality of the LIPSS in terms of regularity of lengths and size of the ripples related to the absorption coefficient of the material at each wavelength, among other parameters like the thickness of the film or the nature of the substrate [15,22,23]. As mentioned before, while LIPSS P3HT films irradiated at 532 nm exhibited selective electrical conductivity, as revealed by C-AFM, those irradiated at 266 nm were comparatively non-conducting [15]. 

### 3.2. Laser-Induced Periodic Surface Structuring on P3HT:PC_71_BM at 266 nm

**Figure 3:**
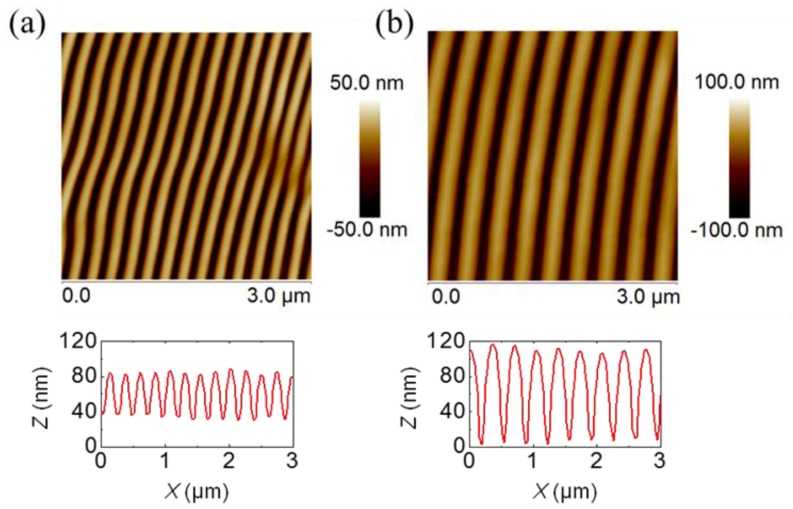
Topography AFM images of LIPSS fabricated with 3600 laser pulses on P3HT:PC_71_BM (1:1) at ambient conditions: (**a**) λ_laser_ = 266 nm with a fluence of 13.5 mJ/cm^2^ and (**b**) λ_laser_ = 532 nm with a fluence of 26 mJ/cm^2^. The height profile along a 3 μm line perpendicular to the ripples is shown below both images. Periods of 225 ± 9 nm and depths of 61 ± 8 nm (λ_laser_ = 266 nm) and of 350 ± 8 nm and depths of 111 ± 9 nm. (λ_laser_ = 532 nm) are obtained.

**Figure 4:**
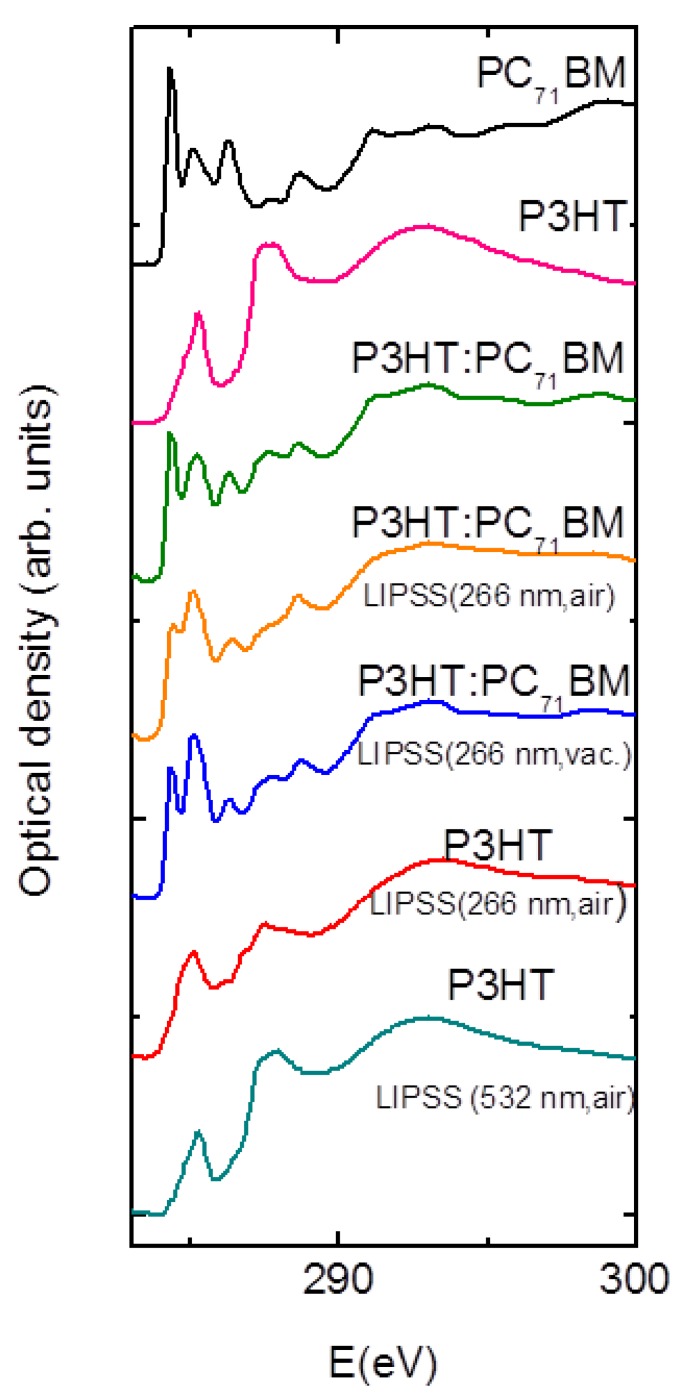
Transmission near-edge X-ray absorption fine structure (NEXAFS) spectra of P3HT:PC_71_BM (1:1) films: Spin-coated (green line), with LIPSS at 266 nm formed upon irradiation at ambient conditions (orange) and in a vacuum (blue). Spectra of P3HT and PC_71_BM thin films are shown as reference (magenta and black, respectively). For the sake of comparison, the spectrum of P3HT, irradiated at 266 and 532 nm in air, are also included (red and dark green lines, respectively).

**Figure A2:**
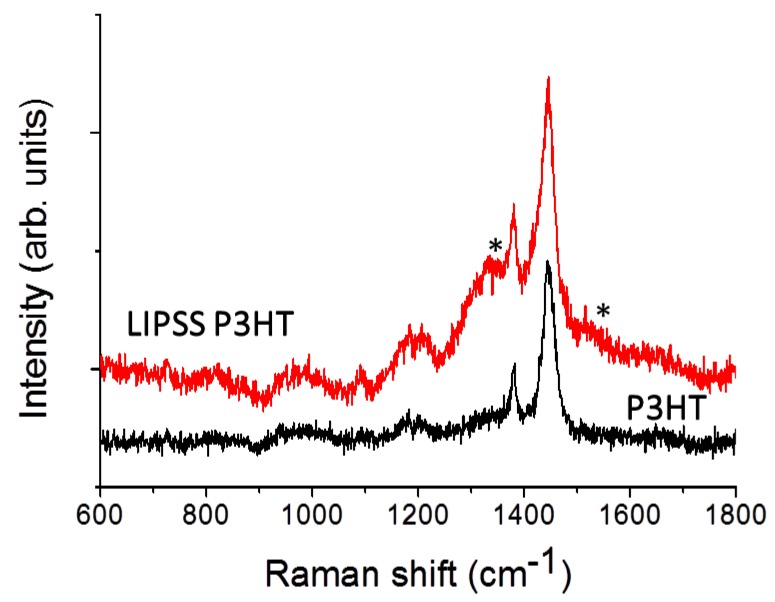
Raman Spectra of P3HT and of the P3HT LIPSS sample after irradiation at 266 nm in ambient conditions. The new bands appearing upon irradiation are indicated by asterisks.

A topography AFM image of LIPSS obtained at ambient conditions on P3HT:PC_71_BM (1:1) at 266 nm with a fluence of 13.5 mJ/cm^2^ and 3600 pulses, is shown in Figure 3a. In order to compare LIPSS obtained on P3HT:PC_71_BM (1:1) at 532 nm, an image corresponding to a sample irradiated with a fluence of 26 mJ/cm^2^ with the same amount of pulses was added (Figure 3b). Contrary to what happens for neat P3HT (Figure 2), for the selected laser conditions, regular LIPSS in P3HT:PC_71_BM (1:1) could be obtained at both laser wavelengths. As mentioned before, in the first approach this effect could be attributed to the absorption, since the PC_71_BM component of the blend absorbed much more than P3HT at 266 nm [17]. P3HT LIPSS samples were electrically non-conducting, which suggested chemical damage by irradiation at UV laser wavelengths. Figure A2 in the Appendix A shows the Raman spectra of P3HT and of the P3HT LIPSS sample after irradiation at 266 nm in ambient conditions. The irradiated sample exhibited the appearance of two additional bands at around 1540 and 1340 cm^−1^. Such an effect has been observed in LIPSS on poly(trimethylene terephthalate) and on polycarbonate bis-phenol A [24]. The new bands could be attributed to the presence of amorphous carbon [25], which suggested that slight carbonization of the film surface appeared upon irradiation at ambient conditions. In order to gain information about the chemical stability of the P3HT:PC_71_BM thin films after laser irradiation at 266 nm, NEXAFS experiments were performed. Figure 4 shows the transmission NEXAFS spectra of a spin-coated P3HT:PC_71_BM (1:1) thin film and of the LIPSS obtained at ambient conditions. The spectra of P3HT and PC_71_BM were included to allow comparison.

The NEXAFS spectrum of the P3HT:PC_71_BM sample with LIPSS at 266 nm obtained at ambient conditions showed a decrease of intensity in two bands. These bands corresponded to the π-band of PC_71_BM at about 284 eV and to the band at 287.5 eV, which corresponded to the σ-bonds [26]. These results could be interpreted as a possible photo-oxidation of the P3HT:PC_71_BM blend after laser irradiation in air at λ_laser_ = 266 nm. This could explain the absence of significant electrical conductivity, as revealed by C-AFM of both P3HT and PC_71_BM after laser irradiation at λ_laser_ = 266 nm. For the sake of comparison, the spectrum of P3HT irradiated at 532 nm in air was included in order to emphasize the absence of significant changes in comparison with that of pristine P3HT.

**Figure 5:**
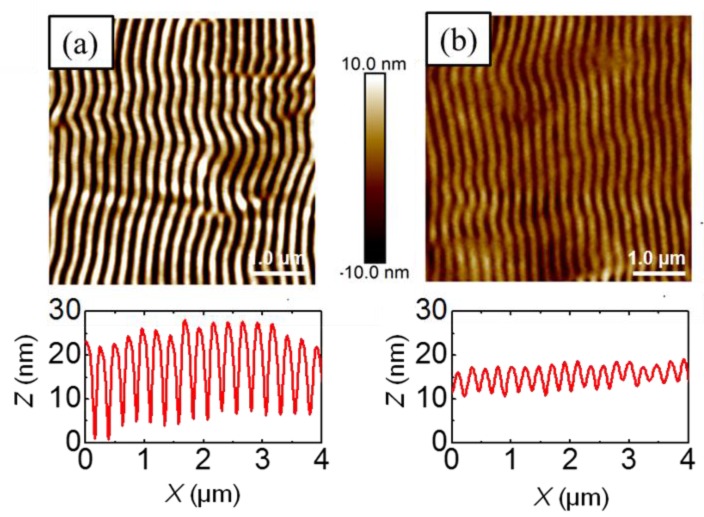
Topography AFM images of LIPSS fabricated at λ_laser_ = 266 nm in vacuum with a fluence of 13.5 mJ/cm^2^ and 3600 pulses on P3HT:PC_71_BM: (**a**) Before and (**b**) after an annealing step for 4 min at 140 °C. In this case, the average period and depth are 230 ± 5 and 8 ± 2 nm, respectively.

Aiming to diminish the effect of possible photo-oxidation at λ_laser_ = 266 nm under ambient conditions, laser irradiation of P3HT:PC_71_BM thin films was carried under vacuum conditions (3 × 10^−2^ mbar). Figure 5a shows the LIPSS morphology after irradiation in vacuum at λ_laser_ = 266 nm. In comparison to irradiation in ambient conditions (Figure 3a), LIPSS of P3HT:PC_71_BM irradiated in a vacuum exhibited an average of similar periods and smaller depths. 

**Figure 6:**
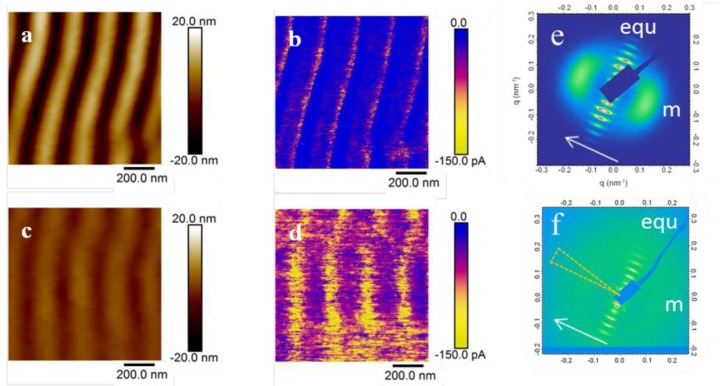
AFM topography of LIPSS on P3HT:P_71_CBM (1:1) fabricated at λ_laser_ = 266 nm in vacuum: (**a**) Before and (**c**) after annealing. Current images of the same samples: (**b**) Before and (**d**) after annealing. Annealing conditions are 4 min at 140 °C. Resonant soft X-ray scattering (RSoXS) patterns at E = 284.2 eV (**e**) before and (**f**) after annealing. The arrows indicate the direction of the ripple axis of the LIPSS. The angular sector indicated in (**f**) refers to the range used for the angular integration of the scattered intensity.

However, the most significant effect was revealed by the NEXAFS experiments (Figure 4). In contrast to the spectra of the samples irradiated on air, LIPSS P3HT:PC_71_BM samples obtained in vacuum exhibited a profile very similar, compared with those of the unstructured P3HT:PC_71_BM, with the exception of the band at 285 eV, which exhibited an increased intensity. Previous works have assigned this band to the sum of the π-bands of P3HT and phenyl groups of PC_71_BM [27,28,29]. Additionally, the intensity of this band was sensitive to the orientation, due to the planarity of the π-bonds. Further angle-resolved NEXAFS work should be done to elucidate the meaning of these changes in the intensity. Nevertheless, it seemed that irradiation at λ_laser_ = 266 nm under vacuum conditions significantly reduced the photo-oxidation. Moreover, under these conditions LIPSS of P3HT:PC_71_BM were structurally stable after thermal annealing conditions (Figure 5b) frequently used for photovoltaic applications. To further verify if the absence of significant photo-oxidation affects the functionality of P3HT:PC_71_BM LIPSS samples irradiated at λ_laser_ = 266 nm, the electrical properties at the nanoscale were analyzed. Figure 6a shows C-AFM electrical current images of P3HT:PC_71_BM thin films nanostructured by LIPSS at λ_laser_ = 266 nm in a vacuum. The current image (Figure 6b) exhibited the characteristic heterogeneous conductivity previously observed in P3HT LIPSS samples irradiated in the visible range (at λ_laser_ = 532 nm) [15], consisting of conducting valleys and non-conducting hills. It is worth emphasizing that P3HT irradiation at λ_laser_ = 266 nm renders non-conducting samples at all. As previously discussed, a decrease of the P3HT crystallinity was the cause of the low conductivity observed in the hills of the LIPSS [15]. After annealing (Figure 6d) the sample presented an overall increase of conductivity, both in the hills and in the valleys of the LIPSS. In order to further characterize the structural changes after laser process and subsequent annealing, we show in Figure 6e,f the RSoXS patterns at E = 284.2 eV before and after annealing, respectively. The obtained information could be envisioned as a Small Angle X-ray Scattering (SAXS) pattern at a particular X-ray energy, referred to as a resonant condition, at which the contrast between the two phases P3HT and PC_17_BM were optimized. As shown in a previous paper, performing X-ray scattering at resonant conditions is quite convenient for these type of samples [17]. The pattern for the P3HT:PC_71_BM LIPSS sample prepared in vacuum consisted of two highly oriented maxima. Firstly, a multiple order equatorial reflection, marked in Figure 6e as “equ”, which was associated to the LIPSS period. Secondly, a meridian one, labeled in Figure 6e as “m”. This has been previously reported as indicating that the PC_71_BM phase is partially segregated, having a characteristic correlation length associated to the scattering maximum, q ≈ 0.39 nm^−1^, corresponding to a d-spacing of ≈16 nm, corresponding to the average distance among the domains [17,30,31,32]. For the LIPSS, the position of the meridian reflection orthogonal to the equatorial one associated to the LIPSS ripple axis, which suggested that the PC_71_BM phase segregation took place within the ripples [17]. For the annealed sample, both the equatorial and meridian maximum became less intense and the meridian one appeared essentially isotropic. This could be interpreted as due to the reduction of the height of the LIPSS in the annealed sample (Figure 2c). While the LIPSS were preserved during annealing, and therefore the equatorial characteristic maxima appeared in the pattern, the PC_71_BM phase segregation decreased and lost its anisotropic character due to the contribution of the non-structured residual film below the LIPSS. 

**Figure 7:**
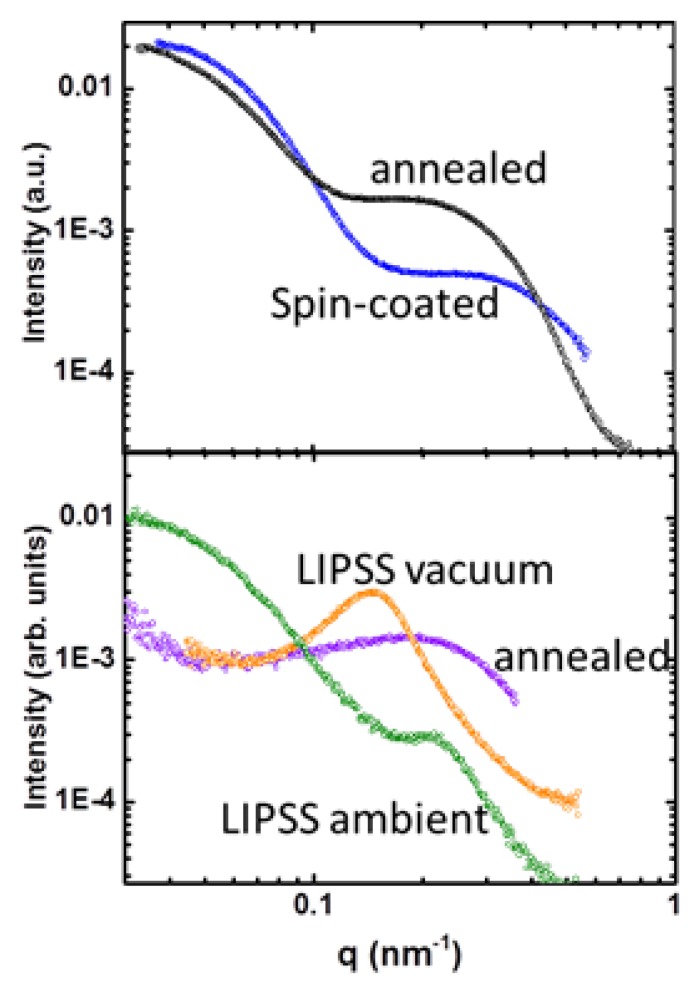
Integrated meridian intensity as a function of the modulus of the scattering vector for the different investigated samples. Upper panel: P3HT:PC_71_BM (1:1) spin-coated film (blue), annealed P3HT:PC_71_BM spin-coated film (black). Bottom panel: LIPSS P3HT:PC_71_BM (1:1) at λ_laser_ = 266 nm in ambient conditions (green), λ_laser_ = 266 nm in vacuum (orange), annealed LIPSS P3HT:PC_71_BM (1:1) (λ_laser_ = 266 nm in vacuum) (purple).

In order to further support this interpretation, we accomplished intensity azimuthal integration of the RSoXS patterns in an angular sector of significance for the meridian maximum indicated in Figure 6f. Figure 7 shows the integrated meridian intensity as a function of the modulus of the scattering vector for the different investigated samples. The original P3HT: PC_71_BM spin-coated film exhibited the characteristic maximum associated to the PC_71_BM phase segregation at q ≈ 0.39 nm^–1^. After annealing of the film phase, the segregation increased as reflected by the shift towards smaller values of the maximum. The LIPSS formation at λ_laser_ = 266 nm in ambient conditions increased phase segregation with respect to the original film, and this effect became more pronounced by irradiating in vacuum. Annealing of the LIPSS P3HT:PC_71_BM sample prepared in vacuum shifted the maximum associated to the PC_71_BM back to value of the initial film, corroborating that due to the reduction of the height of the ripples the residual unstructured film became significant.

## 4. Discussion

**Figure 8:**
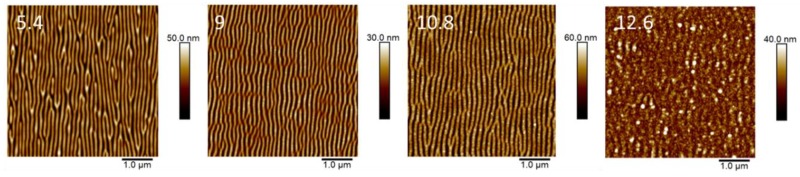
AFM topography images of LIPSS on PC_71_BM thin films at an irradiation wavelength of 266 nm with 3600 pulses as a function of fluence (mJ/cm^2^) labeled on the top left corner.

Our results clearly suggested that low-order LIPSS fabricated in P3HT at 266 nm (Figure 2) could be improved by adding PC_71_BM to form a blend. In this case, ordered LIPSS appeared in P3HT:PC_71_BM during irradiation at 532 nm (Figure 3). In the first approach, differences in the quality of the structures—in terms of the regularity of lengths and the sizes of the ripples—could be related to the absorption coefficient of the material at each wavelength. However, other aspects, like film thickness, roughness and the nature of the substrate, could play an important role [23]. In principle, the higher absorption coefficient of P3HT at 532 nm in relation to that at 266 nm, contributed to the formation of better-ordered ripples irradiating at 532 nm than those observed irradiating at 266 nm. However, the formation of LIPSS in polymer materials with low absorption coefficients has been reported [23,33]. Therefore, it was more likely that the modification of the overall absorption coefficient of the P3HT:PC_71_BM induced by the fullerene component might not be the only synergistic reason to account for the observed improvement of the LIPSS quality of the blends. Previous reports have shown that illumination of PC_61_BM by a UV non-pulsed diverging laser beam can produce micron size patterns [34]. In addition, by using a nanosecond-pulsed laser in the visible wavelength range, we have produced LIPSS on PC_71_BM [35]. In order to gain further information about the synergistic effect of PC_71_BM in the LIPSS formation in P3HT:PC_71_BM (1:1) blends, we accomplished laser irradiation experiments in PC_71_BM thin films in the UV range (λ_laser_ = 266 nm). Figure 8 shows AFM topography images, taken on the center of the laser spot, of PC_71_BM irradiated with 3600 pulses at λ_laser_ = 266 nm in ambient conditions for different fluences. 

**Figure 9:**
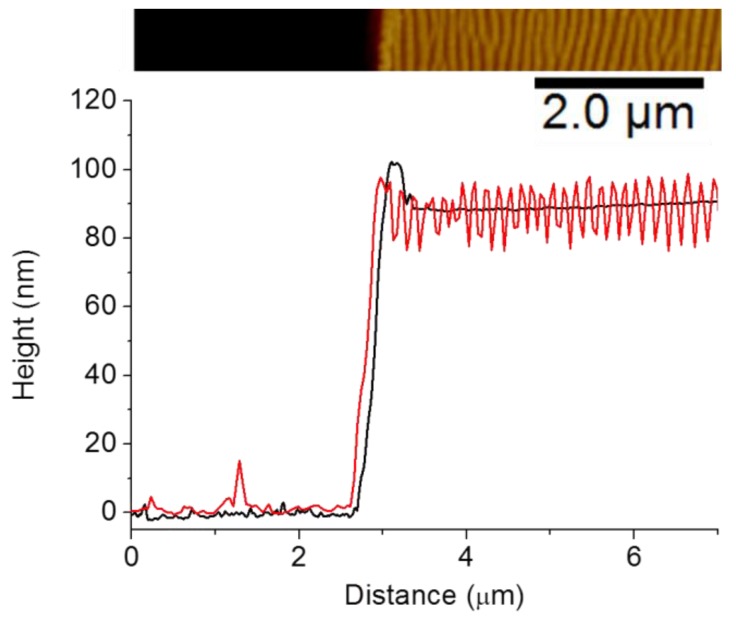
Height profiles of the spot central part after removal with a razor blade part of: PC_71_BM film irradiated at λ_laser_ = 266 nm (3600 pulses, F = 9.0 mJ/cm^2^) (red line) and initial spin-coated PC_71_BM film (black line). The upper inset shows the AFM height image of the LIPSS sample where the profile was calculated. The darker zone on the left corresponds to the part which has been removed by the razor blade.

Regular LIPSS appear on PC_71_BM thin films with periods of about 140 ± 20 nm and depths of about 30 ± 5 nm in a certain range of fluences between 5 and 10 mJ/cm^2^, above which (12.6 mJ/cm^2^) ablation features are detected. A typical LIPSS profile of these samples, taken perpendicular to the ripple after the removal of part of the film with a razor blade is shown in Figure 9. The left part of the data corresponded to the surface of the silicon substrate after the removal of the material by the blade. The corresponding profile for the initial spin-coated PC_71_BM was also presented for comparison. In this case, the difference in height between the flat background on the left and the upper plateau on the right corresponded to the thickness of the spin-coated PC_71_BM film, which is about 90 nm. The superposition of the height profiles of both the initial spin-coated, and that of the irradiated one, indicated that LIPSS formation proceeded on the PC_71_BM surface by a redistribution of the material into hills and valleys. 

These results clearly indicated that PC_71_BM itself could develop quite homogeneous LIPSS by illumination of spin-coated films, with nanosecond pulsed lasers with UV wavelengths (266 nm). 

## 5. Conclusions

Ordered LIPSS could be fabricated in P3HT:PC_71_BM by using wavelengths in the UV range (266 nm), in contrast to the case of neat P3HT. The higher absorption coefficient of PC_71_BM, which has a maximum in its UV–Visible absorption spectrum around 266 nm, enhances the overall absorption of the blend. Our results showed that PC_71_BM is capable of developing homogeneous LIPSS by laser irradiation in the UV range. Therefore, the synergistic effect of this fullerene on the LIPSS formation in P3HT:PC_71_BM (1:1) seemed to be not only due to the overall increment of the absorption of the blend, but also due to the templating effect for LIPSS formation of the PC_71_BM itself. The LIPSS formation at ambient conditions in this wavelength range led to chemical modification of both P3HT and PC_71_BM, which rendered to non-conducting samples. Irradiation of the blends in vacuum significantly reduced radiation damage at λ_laser_ = 266 nm, rendering to the characteristic electrical conductivity pattern observed in P3HT LIPSS samples irradiated in the visible range. The exploitation of this effect in other polymers could potentially be useful in order to obtain LIPSS nanostructures in low absorbing polymers.
